# From evidence to action: bridging the implementation gap for health technology assessment in India

**DOI:** 10.3389/ijph.2026.1610379

**Published:** 2026-10-22

**Authors:** Shuvarthi Bhattacharjee, Neha Chanana, Prakamya Gupta, Swarnali Sarkar, Balu Venugopal, Krushna Chandra Sahoo, Oshima Sachin, Kayala Venkata Jagadeesh, Shankar Prinja, Kavitha Rajsekar

**Affiliations:** 1 Department of Health Research, Ministry of Health and Family Welfare, Government of India, New Delhi, India; 2 Department of Community Medicine and School of Public Health, Postgraduate Institute of Medical Education and Research, Chandigarh, India

**Keywords:** health promotion and education, health systems, health technology assessment (HTA), implementation and dissemination, India

Health technology assessment (HTA) is increasingly recognised as a cornerstone of evidence-informed priority setting in India, supporting universal health coverage and efficient resource allocation [1, 2]. However, the value of HTA depends on its translation into policy and practice. In decentralised pluralistic health systems, variability in procurement, financing, and programme delivery can limit the consistent adoption of cost-effective interventions and reduce HTA impact [3].

The experience in India reflects this challenge. Since its establishment in 2017, Health Technology Assessment India (HTAIn) has produced several assessments to inform decisions across diagnostics, devices, pharmaceuticals, and public health programmes [2, 4]. These assessments have informed price negotiations, health benefit package revisions, and adoption of cost-effective interventions in tuberculosis, cancer, and maternal and child health [5, 6]. Consistent with these impacts, a recent study reported substantial returns on investment from HTA-informed interventions in India, ranging from 8.5:1 to 3,906:1 [7]. However, uptake of HTAIn-recommended technologies across States and Union Territories in India remains variable because of differences in institutional capacity, procurement processes, and administrative priorities [8, 9].

One of the constraints during the early phase of HTAIn has been the relatively weak structured pathways for translation of national HTAIn outputs to subnational action. Firstly, dissemination of HTA findings and recommendations has often been episodic, limiting visibility on implementation status. Secondly, limited availability of HTAIn-recommended technologies on procurement platforms or government e-marketplaces for procurement by payer agencies constrains uptake despite favourable evidence. Thirdly, field-level feasibility concerns affected implementation of HTAIn-recommended technologies within population-wide health programmes. Finally, the budget impact of cost-effective technologies also affected the adoption of these technologies, as demonstrated by the phased adoption of pangenotypic sofosbuvir-velpatasvir for hepatitis C in resource-constrained settings [10]. Collectively, these findings underscore the importance of moving beyond evidence generation towards active implementation support for HTAIn-recommended technologies.

Accordingly, HTAIn has undertaken several initiatives to address the identified gaps. These include constitution of State HTA committees, designation of HTA Nodal Officers by States who serve as the primary liaison between the HTAIn Secretariat and State governments and finally, culminating in the launch of the *A*ccelerating *D*issemination and *O*perational *P*romotion of HTAIn-recommended *T*echnologies (ADOPT) initiative. The ADOPT initiative was introduced as a structured engagement mechanism between national and state stakeholders to strengthen knowledge dissemination and cross-learning on HTAIn-recommended technologies.

So far, Government of Punjab, Haryana, Tamil Nadu and Gujarat have constituted HTA committees. Between May 2025 and July 2026, 50 weekly ADOPT virtual sessions involving State HTA nodal officers, State programme officers and representatives from States and Union Territories were conducted, with an average attendance of approximately 60 participants per session. Each ADOPT virtual session featured a principal investigator sharing findings and recommendations from an HTA study, with discussions focused on clinical effectiveness, value for money, and implementation considerations. These sessions enabled the States to assess the relevance, affordability, and feasibility of adopting recommended health technologies within their local contexts. Consistent with recent evidence on stakeholder dialogues, these engagements served as structured platforms for translating research evidence into implementation-relevant discussions among policymakers and programme stakeholders [11].

Complementing these regular engagements, ADOPT also convened a national sensitisation meeting attended by 134 participants from 28 States and 8 Union Territories. This high-level engagement forum involved senior state health policymakers, administrators and programme officials, whereby HTA findings were disseminated, and dialogue was facilitated on implementation experiences, barriers, and context-specific adaptations across health technologies. Finally, the HTAIn has established five regional ADOPT Centres to support stakeholder engagement, dissemination of HTA evidence, integration of HTA evidence in State-level planning processes, and documentation of post-implementation experiences to facilitate the adoption, scale-up, and refinement of HTAIn-recommended technologies.

The ADOPT experience highlights that implementation of HTA is not a linear process but is shaped by system-level constraints, consistent with established implementation science frameworks. Accordingly, effective uptake of technologies depends not only on the quality of the evidence but also on the capacity of the health systems that support its use. Governance arrangements, stakeholder engagement platforms, institutional capacity, and mechanisms for cross-learning are therefore critical determinants of successful implementation [12–14]. Additionally, where HTA was embedded upstream in programme planning, its influence extended beyond evidence generation to implementation and better resource allocation. This was evident in the tuberculosis control and sickle cell elimination programmes, where HTA-informed procurement decisions generated estimated savings of USD 195 million and USD 5.5 million, respectively, demonstrating the value of integrating HTA into routine programme and financing processes [4, 6]. Another example of the application of HTA evidence in health benefit package design and pricing decisions is India’s government-funded health insurance programme, PM-JAY [5, 9, 15, 16].

These insights are relevant beyond India. Similar patterns of decentralisation exist across Southeast Asia, where subnational entities play a significant role in health service delivery. Strengthening HTA systems in the Southeast Asia therefore requires equal attention to the “last mile” of evidence use. First, structured stakeholder engagement platforms are needed to connect stakeholders generating evidence and policymakers, thereby facilitating dialogue that supports contextual interpretation and implementation pathways.

Second, HTA must be embedded within routine policy and health financing processes. Integrating HTA into programme planning, procurement decisions, and health benefit package design can enable more predictable and accountable adoption of health technologies, as demonstrated through its use in schemes such as tuberculosis control, sickle cell elimination programmes and AB-PMJAY [4, 6]. Without such institutionalisation, the role of HTAIn remains advisory rather than actionable.

Third, strengthening the subnational capacity of the States to use HTA evidence for effective planning, procurement, and adoption of quality and affordable health technologies is crucial for moving research evidence beyond knowledge generation towards programme implementation and real-world impact. Discussions during ADOPT sessions and State HTA committee meetings indicated that differences in technical capacity, availability of field implementation data, and procurement readiness influenced the extent to which States were able to adopt HTAIn recommendations. Addressing these systemic gaps will require sustained investments in developing a cadre of trained policymakers, programme managers, HTA nodal officers, and health administrators capable of interpreting and applying HTA evidence in routine decision-making. HTAIn and HTA resource centres have already initiated capacity strengthening efforts through tailored HTA training workshops, masterclasses from experts and online courses, which can serve as a foundation for further institutionalisation of HTA within routine health system decision-making. Additionally, the recently established ADOPT Centres can play an important role in strengthening implementation capacity of HTAIn recommended technologies by identifying systemic bottlenecks, facilitating peer learning, and supporting States and Union Territories in the consistent adoption of HTAIn-recommended technologies [2].

As Southeast Asia advances towards universal health coverage, the challenge lies increasingly in translating evidence into policy and practice. Within this broader ecosystem, HTAIn has implemented a comprehensive adoption related intervention package. This includes State sensitisation meetings, Designation of HTA Nodal Officers, capacity-building activities, simpler communication of HTA evidence through policy briefs, integration of HTA recommendations into State Programme Implementation Plans, establishment of ADOPT Centres, and the ADOPT discussion series. Collectively, these complementary interventions have strengthened stakeholder engagement, evidence dissemination, cross-State learning, and uptake of HTAIn-recommended technologies.
